# Economic burden of propionic acidemia in the United States: a claims-based study

**DOI:** 10.1186/s13023-025-03836-8

**Published:** 2025-06-11

**Authors:** Sue Perera, Geetanjoli Banerjee, Erin E. Cook, Fan Mu, Mu Cheng, Adina Zhang, Jessie Jie Lan, Lin Zou, Vanja Sikirica

**Affiliations:** 1 Moderna Therapeutics, Inc, 325 Binney St, Cambridge, MA 02142 USA; 2 Analysis Group, Inc., 111 Huntington Avenue, 14th floor, Boston, MA 02199–7668 USA; 3 Moderna Therapeutics, Inc, 5 Vaughn Dr, Princeton, NJ 08540 USA

**Keywords:** Age, Economic burden, Healthcare costs, Healthcare resource utilization, Metabolic decompensation events, Propionic academia

## Abstract

**Background:**

There is currently no prior literature evaluating the economic burden of propionic acidemia (PA) in the US. This study evaluated the healthcare resource utilization (HRU) and expenditures associated with PA, overall and stratified by age.

**Methods:**

The IQVIA PharMetrics^®^ Plus Claims database was used to identify patients with PA and matched (1:1) non-PA control individuals, who were stratified into 0–2, 3–6, 7–12, 13–17, and 18 + years age strata. All-cause HRU and costs were compared between the 2 cohorts by age stratum; PA-related HRU/costs were described for patients with PA.

**Results:**

Among 230 paired observations across age strata, patients with PA had significantly higher all-cause HRU per-person-year (PPY) than control individuals. Patients with PA had 0.47–2.31 inpatient admissions PPY compared to 0.00-0.17 for control individuals (rate ratio: 10.36–78.55, all *p* < 0.001). Patients with PA had 2.23–4.46 times more emergency room visits and 1.89–8.21 times more outpatient visits than control individuals as assessed by rate ratios. Patients with PA incurred significantly higher annualized all-cause total healthcare costs than control individuals, with the highest difference in the 0–2 year old ($205,883) and the lowest in the 7–12 year old age stratum ($20,168; both *p* < 0.001). Among patients with PA, annualized mean PA-related total medical costs were $38,724 overall; inpatient admissions accounted for most costs ($33,575). Patients with PA who experienced metabolic decompensation events (MDEs) had higher HRU and costs than those without MDEs.

**Conclusions:**

Patients with PA, with or without MDEs, had significantly increased HRU and costs than matched controls without PA. Economic burden, largely driven by hospitalizations, was significantly higher among patients with PA than control individuals across all pediatric and adult age strata.

**Supplementary Information:**

The online version contains supplementary material available at 10.1186/s13023-025-03836-8.

## Background

Propionic acidemia (PA) is a life-threatening, inherited metabolic disorder characterized by a deficiency in the enzyme propionyl-coenzyme A (CoA) carboxylase, resulting in reduced catabolism of branched-chain amino acids and accumulation of toxic metabolites like propionic acid and 2-methylcitrate. [1–3] As a rare disease, PA has an estimated birth prevalence of ≤ 1.22 per 100,000 newborns in the United States (US). [1].

The onset of PA is most often observed during the neonatal period or in early infancy, with symptoms such as poor feeding, lethargy, rash, seizures, and difficulty breathing. [4] Rarely, patients may remain asymptomatic until later in life. [5] Symptoms present in older children and adults may include growth retardation, cognitive impairment, cardiomyopathy, pancreatitis, and neurological complications like basal ganglia lesions. [5, 6] Notably, episodes of life-threatening acute metabolic decompensation events (MDEs) may occur when patients are exposed to catabolic stressors like infection, fever, acute trauma, and stress. [2] While MDEs can range in severity, the most serious instances can result in coma, multi-organ failure, and even death. [2].

Despite the life-threatening nature of MDEs, there are currently no approved therapies available to address the underlying enzymatic deficiency of PA. Management of PA centers around medical nutrition therapy to restrict dietary protein and thus reduce the accumulation of toxic metabolites. [7] Additionally, antibiotics and supplements (e.g., biotin, L-carnitine) are commonly used to achieve and maintain metabolic stability. [3, 7] In patients with severe disease and frequent MDEs, liver transplantation may be considered to restore hepatic enzymatic activity. [3].

Given the need for long-term nutritional support and recurrent hospitalizations for MDEs, healthcare resource utilization (HRU) and expenditures are likely substantial for patients with PA. [8] Indeed, in a single-center study of electronic health records (EHR) from 13 patients with PA at the Mayo clinic, nearly half had at least 1 inpatient hospitalization over a median of 2 years of care. [9] However, to the best of our knowledge, there is currently no prior literature evaluating the economic burden of PA in the US. As such, the current study was conducted to address this evidence gap by comparing the HRU and healthcare costs between patients with and without PA, with stratifications by age to characterize natural progression and changes throughout life. Additionally, outcomes were further assessed among patients with PA, with or without MDEs, as well as before or after the onset of the COVID-19 pandemic.

## Methods

### Data source

Administrative healthcare claims data from 10/1/2015- 6/20/2022 from the IQVIA PharMetrics^®^ Plus Claims database were used. Over 190 million unique beneficiaries are covered by the IQVIA PharMetrics^®^ Plus Claims database, of which over 150 million are covered by both medical and pharmacy plans. Data are deidentified and comply with the patient confidentiality requirements of the Health Insurance Portability and Accountability Act (HIPAA); therefore, no reviews by an institutional review board were required.

### Study design and population

Details regarding the study design and study population were described previously. In brief, a retrospective, observational, matched cohort study design was used to compare economic outcomes between patients with PA and their matched non-PA control individuals within each of the following 5 age strata: 0–2 years, 3–6 years, 7–12 years, 13–17 years, and 18 + years.

The study periods were defined individually for each age stratum. For the first age stratum, the index date was defined as the date of the first observed PA diagnosis for patients with PA, or the date of the first medical or pharmacy claim for non-PA matched control individuals. For all subsequent age strata, the index date for patients in both cohorts was defined as the earliest of the first day in the respective age stratum or the start of the enrollment period. The observation period for each age stratum was defined as the time between the index date to the earliest of end of health plan enrollment, end of age stratum, or end of data availability.

#### Sample selection

Patients were included in the study if they met the following inclusion criteria: (1) either had (PA cohort) or did not have (control cohort) a diagnosis of PA (International Classification of Diseases, Tenth Revision, Clinical Modification [ICD-10-CM]: E71.121) on or after October 1, 2015, (2) had no missing age information, and (3) had ≥ 6 months of continuous eligibility after the index date in any age stratum.

Patients in the PA and control cohorts were exactly matched within their age stratum on a 1:1 ratio based on the variables described previously.

### Study outcomes

Study outcomes evaluated during the observation period included HRU and healthcare costs. All-cause and PA-related HRU included inpatient admissions, outpatient visits, home health visits, skilled nursing facility (SNF) visits, and emergency department (ED) visits. PA-related HRU was only evaluated among patients with PA and was defined as encounters with a diagnosis code for PA, hyperammonemia, or metabolic acidosis in any position.

All-cause healthcare costs included total healthcare costs, comprising total pharmacy costs and total medical costs (i.e., costs for inpatient admissions, outpatient visits, home health visits, SNF visits, and ED visits). Costs were inflation-adjusted to 2022 US dollars based on the medical care component of the Consumer Price Index. [10] PA-related medical costs were also examined for patients with PA; PA-related pharmacy costs were not evaluated due to difficulties in ascertaining PA-related pharmacy claims.

### Statistical analysis

SAS Enterprise 7.1 software (SAS Institute, Cary, NC, USA) and R version 4.0.3 were used for all statistical analyses.

Patient demographics at index date and clinical characteristics during the 6 months following the index date were described for patients with PA and matched non-PA control individuals within each age stratum using means and standard deviations (SDs) for continuous variables and frequencies and proportions for categorical variables. Characteristics were compared between the two cohorts using Chi-square tests for categorical variables and Wilcoxon signed rank tests for continuous variables.

The proportion of patients with any all-cause or PA-related HRU during the observation period was described using counts and proportions and compared using Chi-squared tests within each age stratum. All-cause and PA-related HRU rates were summarized on a per-person-year (PPY) basis during the entire observation period in each age stratum (i.e., total number of encounters divided by the total person-years of observation). Comparison of all-cause HRU rates patients with PA and non-PA matched control individuals was conducted using generalized estimating equations (GEEs) with a Poisson distribution and robust sandwich estimators to account for matching. Rate ratios (RRs) and 95% confidence intervals (CIs) were reported.

Annualized all-cause healthcare costs during the observation period were described for patients with PA and matched non-PA control individuals within each age stratum using means and SDs, and compared using Wilcoxon signed-rank tests. Mean annualized cost differences between the two cohorts, along with SDs, were reported.

A nominal two-sided alpha error of 0.05 was used to determine statistical significance for all comparative analyses.

### Subgroup analyses

For the subgroup analyses, patients with PA were further stratified into subgroups based on presence or absence of MDEs at any time during the observation period, where a MDE was defined as a medical claim in an inpatient setting with a diagnosis code for hyperammonemia and/or metabolic acidosis in any position. Additionally, patients with PA were also stratified by observation time occurring before or after the start of the COVID-19 pandemic (i.e., March 2020). Patients with observation time both before and after the start of the COVID-19 pandemic contributed time to both cohorts, respectively.

All comparisons were replicated between patients with MDEs versus those without any MDEs, as well as between patients with MDEs versus their matched non-PA control individuals, and between patients without MDEs versus their matched non-PA control individuals. Furthermore, all comparisons were replicated between patients with PA, before and after the onset of the COVID-19 pandemic.

## Results

### Study population

The study included 191 patients with PA and 230 age stratum–matched non-PA control individuals, contributing 230 paired observations across all age strata. There were 32 matched pairs in the 0–2 years age stratum, 32 in the 3–6 years age stratum, 36 in the 7–12 years age stratum, 24 in the 13–17 years age stratum, and 106 in the 18 + years age stratum.

Patient characteristics have been described previously. Briefly, patients with PA and matched non-PA control individuals had similar demographic characteristics. Among all patients with PA, mean age at first observed PA diagnosis in available data was 24.8 years, 49.7% were female, and 91.1% had commercial insurance or were self-insured. Compared to matched non-PA control individuals, patients with PA were more likely to experience comorbidities (e.g., neurologic/nervous system, growth complications, metabolism-related conditions) and PA-related symptoms (e.g., anorexia/failure to feed, vomiting, metabolic acidosis).

The mean (median) duration of observation for all patients with PA was 2.65 (2.04) years.

### HRU among patients with PA compared with matched non-PA control individuals

### All-cause HRU

Compared with matched non-PA control individuals, patients with PA had significantly higher rates of all-cause HRU across all age strata.

**Fig. 1:**
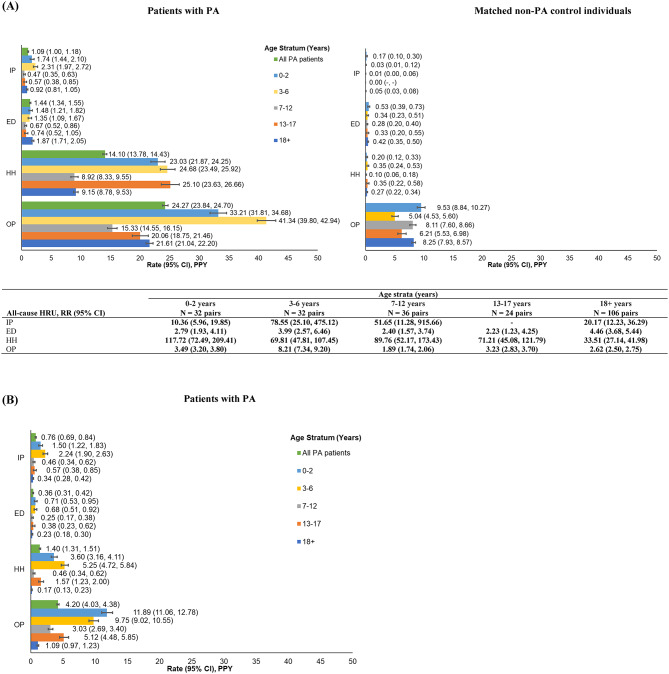
Rates of (**A**) all-cause HRU among patients with PA and matched non-PA control individuals, and (**B**) PA-related HRU among patients with PA. Values in boldface indicate statistically significant difference between patients with PA and control individuals within that age stratum (*p* < 0.05). Abbreviations: CI, confidence interval; ED, emergency department; HH, home health; HRU, healthcare resource utilization; IP, inpatient; OP, outpatient; PA, propionic acidemia; PPY, per-person-year; RR, rate ratio

During the observation period and across age strata, a higher proportion of patients with PA had any inpatient admissions compared with matched non-PA control individuals (33.3-65.6% vs. 0.0-34.4%; all *p* < 0.05; Table 1). Across age strata, rates of all-cause inpatient admissions were 10.36 to 78.55 times higher among patients with PA (range of 0.47–2.31 PPY) than matched non-PA control individuals (range of 0.00-0.17 PPY; all *p* < 0.001; Fig. 1A). On average, across age strata, patients with PA were hospitalized for 3.7 (SD: 1.8) to 14.0 (15.7) days for each stay, while the matched non-PA control individuals were hospitalized for 3.6 (2.0) to 8.0 (SD not evaluable based on one patient with hospitalization stay) days for each stay (data not presented).

**Table 1:** Proportion of patients with all-cause and PA-related HRU during the observation period

		Age strata									
	All patients with PA	0–2 years *N* = 32 pairs		3–6 years *N* = 32 pairs		7–12 years *N* = 36 pairs		13–17 years *N* = 24 pairs		18 + years *N* = 106 pairs	
	*N* = 191	PA	Controls	PA	Controls	PA	Controls	PA	Controls	PA	Controls
**All-cause HRU**,** n (%)**											
IP	110 (57.6%)	**21 (65.6%)**	**11 (34.4%)**	**17 (53.1%)**	**2 (6.3%)**	**14 (38.9%)**	**1 (2.8%)**	**8 (33.3%)**	**0 (0.0%)**	**64 (60.4%)**	**11 (10.4%)**
ED	143 (74.9%)	22 (68.8%)	21 (65.6%)	**20 (62.5%)**	**10 (31.3%)**	25 (69.4%)	16 (44.4%)	16 (66.7%)	10 (41.7%)	75 (70.8%)	63 (59.4%)
HH	110 (57.6%)	**22 (68.8%)**	**11 (34.4%)**	**19 (59.4%)**	**1 (3.1%)**	**22 (61.1%)**	**4 (11.1%)**	**15 (62.5%)**	**2 (8.3%)**	**55 (51.9%)**	**21 (19.8%)**
OP	190 (99.5%)	32 (100.0%)	32 (100.0%)	32 (100.0%)	32 (100.0%)	36 (100.0%)	35 (97.2%)	24 (100.0%)	23 (95.8%)	104 (98.1%)	101 (95.3%)
SNF	5 (2.6%)	0 (0.0%)	0 (0.0%)	1 (3.1%)	0 (0.0%)	0 (0.0%)	0 (0.0%)	0 (0.0%)	0 (0.0%)	4 (3.8%)	1 (0.9%)
**PA-related HRU**,** n (%)**											
IP	92 (48.2%)	19 (59.4%)	-	17 (53.1%)	-	13 (36.1%)	-	8 (33.3%)	-	49 (46.2%)	-
ED	74 (38.7%)	11 (34.4%)	-	15 (46.9%)	-	15 (41.7%)	-	9 (37.5%)	-	32 (30.2%)	-
HH	36 (18.9%)	12 (37.5%)	-	13 (40.6%)	-	8 (22.2%)	-	6 (25.0%)	-	8 (7.6%)	-
OP	133 (69.6%)	25 (78.1%)	-	29 (90.6%)	-	31 (86.1%)	-	16 (66.7%)	-	60 (56.6%)	-
SNF	0 (0.0%)	0 (0.0%)	-	0 (0.0%)	-	0 (0.0%)	-	0 (0.0%)	-	0 (0.0%)	-

Values in boldface indicate statistically significant difference between patients with PA and control individuals within that age stratum (*p* < 0.05)

**Abbreviations**: ED, emergency department; HH, home health; HRU, healthcare resource utilization; IP, inpatient; OP, outpatient; PA, propionic acidemia; SNF, skilled nursing facility

During the observation period and across age strata, a numerically higher proportion of patients with PA had any ED visits compared with matched non-PA control individuals, though this was not statistically significantly different (62.5-70.8% vs. 31.3-65.6%; Table 1). Across age strata, rates of all-cause ED visits were 2.23 to 4.46 times higher among patients with PA (range of 0.67–1.87 PPY) than matched non-PA control individuals (range of 0.28 to 0.53 PPY; all *p* < 0.05; Fig. 1A).

During the observation period and across age strata, a higher proportion of patients with PA had any home health visits compared with matched non-PA control individuals (51.9-68.8% vs. 3.1-34.4%; all *p* < 0.05; Table 1). Across age strata, rates of all-cause home health visits were 33.51 to 117.72 times higher among patients with PA (range of 8.92–25.10 PPY) than matched non-PA control individuals (range of 0.10–0.35 PPY; all *p* < 0.001; Fig. 1A).

During the observation period and across age strata, almost all patients with PA and matched non-PA control individuals had an outpatient visit (98.1-100.0% vs. 95.3-100.0%; Table 1). However, across age strata, rates of all-cause outpatient visits were 1.89 to 8.21 times higher among patients with PA (range of 15.33–41.34 PPY) compared to matched non-PA control individuals (range of 5.04–9.53 PPY; all *p* < 0.001; Fig. 1A).

Among patients with PA, the rates of all-cause HRU were typically higher in younger patients than in older patients (Fig. 1A). Rates of inpatient admissions, ED visits, and outpatient visits were highest in the 0–2 years and 3–6 years age strata, and lowest in the 7–12 years age stratum. Patients with PA aged 13–17 years had the highest rate of home health visits. HRU rates were slightly higher among adult patients (aged 18 + years) with PA compared to adolescents across different types of HRU, except for home health visits.

#### PA-related HRU

PA-related HRU was common among patients with PA, with 48.2% having a PA-related inpatient admission, 38.7% having a PA-related ED visit, 18.9% having a PA-related home health visit, and 69.6% having a PA-related outpatient visit during the observation period. No patients had a PA-related SNF visit (Table 1).

Across age strata, patients with PA had 0.34 to 2.24 PA-related inpatient admissions PPY, 0.23 to 0.71 PA-related ED visits PPY, 0.17 to 5.25 PA-related home health visits PPY, and 1.09 to 11.89 PA-related outpatient visits PPY (Fig. 1B). On average, patients with PA were hospitalized for 3.8 (SD: 1.8) to 15.7 (16.3) days for each PA-related inpatient stay (data not presented).

Similar to all-cause HRU, rates of PA-related HRU were higher in younger patients than in older patients (Fig. 1B). Rates of PA-related inpatient admissions and home health visits were highest in the 3–6 years age stratum, while rates of PA-related outpatient visits and ED visits were highest in the 0–2 years age stratum. Adult patients had the lowest PA-related HRU rates.

### Healthcare costs among patients with PA compared with matched non-PA control individuals

#### All-cause costs

**Fig. 2:**
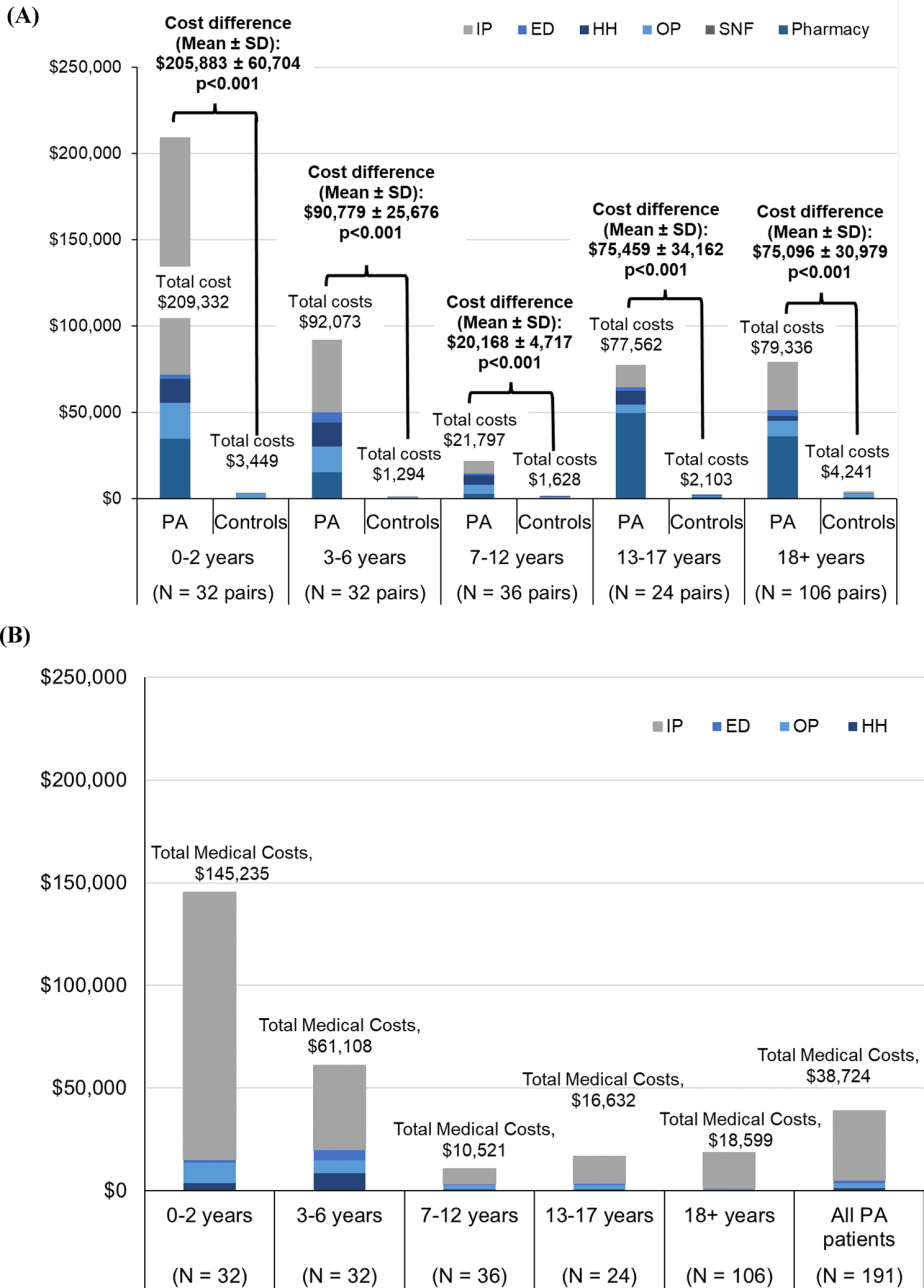
Annualized (**A**) all-cause healthcare costs among patients with PA and matched non-PA control individuals, and (**B**) PA-related medical costs among patients with PA [1, 2]. Abbreviations: ED, emergency department; HH, home health; IP, inpatient; OP, outpatient; PA, propionic acidemia; SD, standard deviation; SNF, skilled nursing facility. **Notes**: [1] Costs were inflation-adjusted to 2022 US dollars based on the medical care component of the Consumer Price Index. [2] PA-related SNF costs were $0 for all patients with PA and across age strata

Compared to matched non-PA control individuals, patients with PA incurred significantly higher mean (SD) annualized all-cause total healthcare costs across all age strata (0–2 years: $209,332 [$343,355] vs. $3,449 [$5,270]; 3–6 years: $92,073 [$145,232] vs. $1,294 [$1,758]; 7–12 years: $21,797 [$28,176] vs. $1,628 [$2,641]; 13–17 years: $77,562 [$167,300] vs. $2,103 [$4,379]; 18 + years: $79,336 [$318,845] vs. $4,241 [$7,970]; all *p* < 0.001; Fig. 2A). Cost differences (SD) were highest in the 0–2 years age stratum ($205,883 [$60,704]) and lowest in the 7–12 years age stratum ($20,168 [$4,717]; both *p* < 0.001).

Among patients with PA, total healthcare costs were highest in the 0–2 years age stratum ($209,332 [SD: $343,355]) and lowest in the 7–12 years age stratum ($21,797 [$28,176]), with a gradual increase towards adulthood (18 + years: $79,336 [$318,845]). Across all age strata, all-cause medical costs accounted for approximately 68% of total all-cause healthcare costs ($60,385 [$136,310] out of $88,523 [$267,258] for all patients with PA), which were driven primarily by inpatient admission costs ($40,621 [$114,420]). The detailed breakdown of all-cause costs is described in Supplemental Table S1.

#### PA-related medical costs

Among all patients with PA, the annualized mean PA-related total medical costs were $38,724 (SD: $123,248) overall, with PA-related inpatient admissions accounting for the majority of costs ($33,575 [$112,079]; Fig. 2B). Similar to all-cause healthcare costs, PA-related total medical costs were highest in the 0–2 years age stratum ($145,235 [$272,189]) and lowest in the 7–12 years age stratum ($10,521 [$22,907]), with a slight increase in adult patients ($18,599 [$60,976]). The detailed breakdown of PA-related costs is described in Supplemental Table S1.

### Economic outcomes in the presence or absence of MDE subgroups

Sixty patients with PA experienced at least one MDE during the study period, while the remaining 131 patients with PA did not experience any MDEs. Patient characteristics for patients with and without MDEs have been described previously. Demographics were similar between patients with and without MDEs. However, patients with MDEs were more likely to have experienced PA-related symptoms within 6 months of the index date than those without MDEs (86.7% vs. 42.8%; *p* < 0.001).

**Fig. 3:**
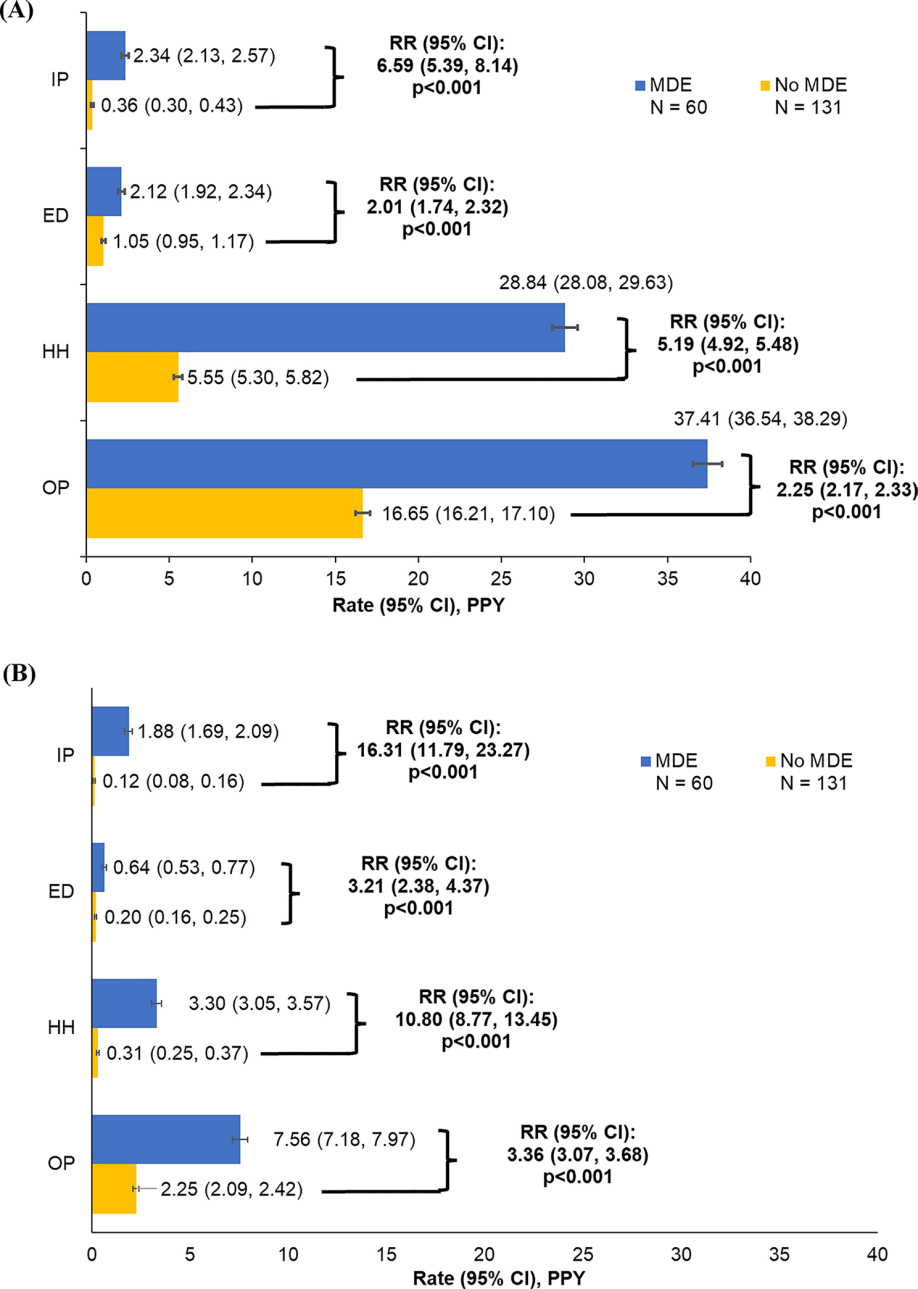
Rates of (**A**) all-cause and (**B**) PA-related HRU among patients with PA, with or without MDEs. Abbreviations: CI, confidence interval; ED, emergency department; HH, home health; HRU, healthcare resource utilization; IP, inpatient; MDE, metabolic decompensation event; OP, outpatient; PA, propionic acidemia; PPY, per-person-year; RR, rate ratio

Patients with MDEs had higher rates of all-cause (Fig. 3A) and PA-related HRU (Fig. 3B) compared to patients without MDEs (all *p* < 0.001). The biggest differences were observed in inpatient admissions, where patients with MDEs had 6.59 times higher all-cause inpatient admission rate and 16.31 times higher PA-related inpatient admission rate compared to those without MDEs. Similar to the main analysis, the rates of all-cause HRU were generally higher in younger patients than in older patients in both the with and without MDEs subgroups (Supplemental Table S2).

**Fig. 4:**
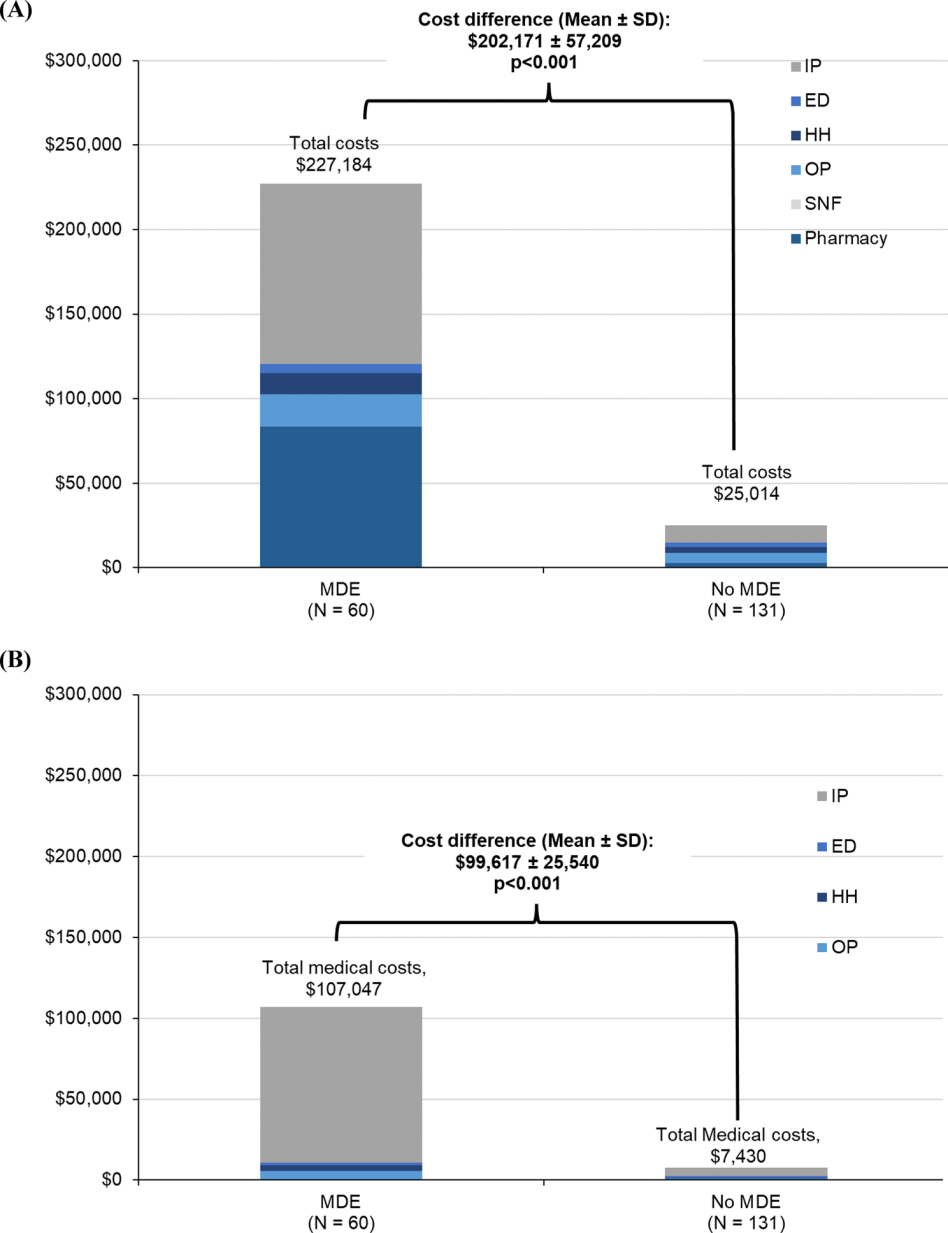
Annualized (**A**) all-cause healthcare costs and (**B**) PA-related medical costs among patients with PA, with or without MDEs [1, 2]. Abbreviations: ED: emergency department; HH: home health; IP: inpatient; MDE: metabolic decompensation event; OP: outpatient; PA: propionic acidemia; SD: standard deviation; SNF: skilled nursing facility. **Notes**: [1] Costs were inflation-adjusted to 2022 US dollars based on the medical care component of the Consumer Price Index. [2] PA-related SNF costs were $0 for all patients with PA and across age strata

In both patient groups, with and without MDEs, inpatient costs accounted for the largest share of total healthcare costs (Supplemental Table S3). Compared to patients without MDEs, annualized all-cause total healthcare costs were higher by $202,171 (SD: $57,209) in patients with MDEs, and annualized PA-related medical costs were higher by $99,617 ($25,540) (both *p* < 0.001; Fig. 4A and B). Inpatient admission costs were the main driver of the total medical cost difference, accounting for 79.6% and 91.8% of the all-cause and PA-related medical cost difference, respectively (mean difference in inpatient admission costs: all-cause: $96,721 [SD: $23,674], PA-related: $91,469 [SD: $23,523]; Supplemental Table S3).

Compared with matched non-PA control individuals, both patients with PA, with or without MDEs, had significantly increased HRU and healthcare costs (Supplemental Tables S4 and Supplemental Table S5). The differences were more pronounced for patients with MDEs.

### Economic outcomes in the before or after COVID-19 onset subgroups

Among 191 patients with PA, 140 had at least 6 months of follow up prior to the onset of the COVID-19 pandemic and 119 had at least 6 months of follow up following the onset of the COVID-19 pandemic.

Patients with PA generally had higher PPY rates of all-cause HRU during the pre-COVID-19 period than post-COVID-19 onset periods (inpatient admissions: 1.30 vs. 0.76; outpatient visits: 25.25 vs. 23.00; home health visits: 15.04 vs. 12.77; ED: 1.62 vs. 1.20; rate data not shown), with consistent patterns for PA-related HRU, except for PA-related home health visits, which appeared to be higher during the post-COVID-19 onset period (Supplemental Table S6; inpatient admissions: 0.94 vs. 0.47; outpatient visits: 4.99 vs. 2.88; home health visits: 0.92 vs. 1.84; ED: 0.44 vs. 0.23; rate data not shown).

All-cause and PA-related healthcare costs were similar between the pre- and post-COVID-19 periods, with a trend towards higher costs during the pre-COVID-19 period (Supplemental Table S7).

## Discussion

To our knowledge, this study represents the first claims-based analysis of the economic burden of PA in the US, utilizing a large sample primarily composed of commercially insured individuals. HRU and healthcare expenditures were significantly higher among patients with PA compared with matched non-PA control individuals across pediatric and adult age strata, with the burden largely driven by inpatient admissions. Among patients with PA, those with MDEs during the observation period had significantly higher HRU and costs compared with those without MDEs. However, a substantial economic burden remained for patients without MDEs when compared to matched non-PA control individuals. Findings from this study underscore the significant economic burden incurred by patients with PA and highlight the unmet need for an effective therapy in this population.

There is limited prior literature evaluating the economic burden of PA in the US. Barman et al. conducted a small, single-center, EHR-based analysis of 13 patients with PA who were treated at the Mayo Clinic between 1998 and 2022 and found that 46% and 39% received care in the inpatient and ED settings, respectively, over a median follow-up of 2 years. [9] In comparison, the proportions observed in the current study (i.e., 57.6% with any inpatient admissions and 74.9% with any ED visits over a median of 2 years) were higher, which potentially reflects the more comprehensive capture of HRU in claims data, as patients may seek healthcare at multiple centers. Additionally, other outcomes like healthcare costs and age stratifications were not examined by Barman et al.

In the present study, patients with PA incurred significantly increased healthcare costs compared to matched non-PA control individuals, with excess annual, all-cause, total healthcare costs ranging from $20,168 to $205,883. This large cost difference was driven by particularly high inpatient costs among patients with PA, which accounted for approximately 46% of the all-cause total healthcare costs. Notably, a substantial portion of inpatient admissions were PA-related; among patients with PA, the rate of all-cause inpatient admissions was 1.09 PPY relative to 0.76 PPY for PA-related inpatient admissions, and approximately 84% of patients with an all-cause inpatient admission had a PA-related inpatient admission. Similarly, 83% of all-cause inpatient costs were PA-related ($33,575 of $40,621) in this study, highlighting the frequent need for costly hospitalizations related to PA management.

After inpatient costs, pharmacy costs comprised the second-largest proportion of all-cause total healthcare costs (32%). Despite this finding, treatment costs are likely underestimated in this study, because PA management generally consists of dietary modifications and nutritional supplementation that may not be captured through claims data. [7] Indeed, daily nutritional support can be quite costly, with low-protein foods costing up to 8 times more than their regular counterparts, and L-carnitine and vitamin B12 supplements costing more than $18,000 per year which are often paid out of pocket, therefore not captured by insurance claims data. [8].

This study further contributes important insight to the limited real-world PA literature by characterizing the economic burden across various age groups. HRU and healthcare costs were generally highest among pediatric patients (i.e., 0–6 years), decreased towards adolescence (i.e., 7–17 years), and increased slightly in adulthood (i.e., 18 + years). This trend is consistent with the clinical findings from a prior analysis of the same patient population by Banerjee et al., where younger patients had the highest rates of MDE, followed by a decline among patients aged 13–17 years, and finally a slight increase among adults. [11] This alignment suggests that the time-varying clinical burden of PA may be associated with corresponding changes in HRU and healthcare costs throughout a patient’s life, thus emphasizing the need for effective treatment that may achieve improved disease control and potentially reduce the lifelong economic burden associated with PA. Additionally, the lower costs for mid-childhood (ages 7–12 years) may reflect a period of relative clinical stability in the natural history of PA. Compared to infants and younger children who often require intensive diagnostic evaluations, feeding support, and management of early MDEs, patients in mid-childhood may experience fewer acute episodes as they and their caregivers become more adept at managing the disease. Conversely, costs begin to rise again in adolescence and adulthood, likely due to the increasing burden of chronic complications such as cardiomyopathy and pancreatitis. While these complications may emerge in later childhood, their severity and associated healthcare needs often escalate with age. For example, Baumgartner et al. reported a median age of cardiomyopathy presentation at 7 years, [12] suggesting that both clinical complexity and economic burden tend to increase beyond mid-childhood. However, given the smaller sample sizes among patients in the non-adult groups (24 to 36 pairs per age group), caution should be used when interpreting patterns between specific age groups.

Home health visits were elevated in patients with PA compared to controls across age groups and may reflect a more proactive, supportive approach to care. While the impact of shifting the type of care received on cost and cost patterns among adult age groups were not examined in this study, further research examining these questions would be meaningful among patients with PA. For example, a shift to home health visits may help prevent more costly hospitalizations over time, potentially reducing the overall economic burden, particularly in older age groups, where clinical complications often require intensive resource use and drive higher costs. Additionally, as PA is a chronic, progressive, multi-organ disease, providing care for these patients requires a large multi-disciplinary team of medical specialists. While describing the specialists seen by patients is an important aspect to further understanding and potentially improving care, it was not the primary goal of the current study, which aims to more broadly characterize the care setting utilized by patients with PA. This is an important research question among patients with PA that future research should address.

As claims data lack the clinical detail necessary to distinguish PA phenotypes or severity at initial onset, subgroup analyses among patients with and without MDEs were conducted to approximate differences in disease severity. In this subgroup analysis, patients with PA and MDEs incurred a larger economic burden than those without MDEs, which may reflect the more complex and costly management of more severe disease in the former subgroup, such as increased use of intensive care unit-level care and changes in medication dosing and routes of administration (e.g., intravenous carnitine). Notably, 91.8% of the PA-related cost difference between patients with and without MDEs was driven by inpatient admission costs, including hospital stays for MDEs, underscoring the significant burden associated with these severe disease episodes. Indeed, Barman et al. found that patients with PA and MDEs were more likely to present with severe complications (e.g., cardiovascular issues and neurologic abnormalities) and require PA-related procedures (e.g., gastronomy tube feedings and hemofiltration) than those without MDEs. [9] Similarly, patients with PA and MDEs had a larger clinical burden, including PA symptoms and comorbidities, than those without MDEs in the study by Banerjee et al. [11].

In addition to the MDE subgroup analysis, this study was the first to assess the impact of the COVID-19 pandemic on the economic burden of PA. Patients with PA generally had higher rates of HRU during the pre-COVID-19 period relative to the post-COVID-19 period, with a corresponding trend towards higher healthcare costs. While reasons for this change were not investigated, the findings may reflect the overall reduction in healthcare-seeking behaviors during the pandemic, [13] or the decreased exposure to MDE triggers like infections as a result of social distancing measures. [14].

### Limitations

As previously mentioned, PA-related pharmacy costs were not evaluated given the lack of PA-indicated and approved medications and broad breadth of use of symptomatic treatments for PA. However, the use of all-cause pharmacy costs captures the overall impact of medication burden. Additionally, data regarding indirect costs (e.g., productivity loss due to caregiving for children with PA) were not available in claims. These costs may contribute substantially to the overall disease burden of PA in addition to the direct medical and pharmacy costs evaluated in the current study. Future research can further delve into the costs and impacts of medications used as well as indirect costs. There was also not enough data to assess the change in economic burden before or after liver transplantation, which is an important driver of disease burden and should be explored in future studies using data sources with more comprehensive information on liver transplantation. Similarly, some elements of care, such as home-based point-of-care testing or services rendered outside the network, may not be fully captured in the claims database, leading to an underestimation of the overall economic burden in this population. It was not possible to definitively assess the burden of PA from disease onset because the ICD-10-CM diagnosis code for PA has only been available since October 2015 and the claims data were subject to left censoring, thus limiting the capture of initial diagnoses. Relatedly, PA-related encounters may have been underestimated if the diagnosis code was not systematically recorded in the associated claims.

The stratified findings should be interpreted with caution given the smaller sample sizes in certain age strata and the subgroup analyses. Moreover, no statistical adjustments were made for the subgroup analyses, which were descriptive comparisons. Lastly, the study sample included patients with commercial insurance which may present with a milder form of disease; therefore, results may not be generalizable to patients with other or no insurance coverage.

## Conclusions

In this first-of-its-kind, US claims-based, retrospective analysis of the economic burden of PA, patients with PA experienced significantly higher HRU and healthcare costs compared with matched non-PA control individuals. This economic burden was more pronounced among pediatric patients aged 0–2 and 3–6 years and adult patients than adolescents aged 7–12 and 13–17 years, and among patients who experienced MDEs than those who did not. These findings highlight the need for novel treatments for PA with effective disease management, which may help to reduce the disease burden in this population. Future research may consider extending the population to include pediatric patients covered by Medicaid, conducting analyses stratified by disease severity, and further examining outcomes such as liver transplant, mortality, or indirect costs incurred by patients and caregivers.

## Electronic supplementary material

Below is the link to the electronic supplementary material.

Supplementary Material 1

## Data Availability

The datasets generated and analyzed during the current study are not publicly available because they were used pursuant to a data use agreement. The data are available through requests made directly to IQVIA.

## Abbreviations

CI: Confidence interval
ED: Emergency department
EHR: Electronic health records
GEE: Generalized estimating equation
HIPAA: Health Insurance Portability and Accountability Act
HRU: Healthcare resource utilization
IP: Inpatient
MDE: Metabolic decompensation event
PA: Propionic acidemia
PPY: Per-person-year
RR: Rate ratio
SD: Standard deviation
SNF: Skilled nursing facility
US: United States
USD: United States dollar

## Declarations

### Previous presentation

Part of the material in this manuscript was presented at ISPOR Europe 2023, held on November 12–15 in Copenhagen, Denmark as a poster presentation.
