# Supplementary material for: Economic burden of propionic acidemia in the United States: a claims-based study
Source: Orphanet J Rare Dis. 2025 Jun 11;20:295. doi: 10.1186/s13023-025-03836-8 (PMC12153125; doi:10.1186/s13023-025-03836-8)
Supplement: Supplementary file 1 — Supplementary Material 1 [file 13023_2025_3836_MOESM1_ESM.docx]

# SUPPLEMENTARY MATERIALS

## Supplemental Table 1. Detailed annualized all-cause and PA-related healthcare costs in the (A) 0-2 and 3-6 years age strata and (B) 7-12, 13-17, and 18+ years age strata^1^

**(A)**

|  | **All patients with PA** | **Age strata (years)** | | | | | |
| --- | --- | --- | --- | --- | --- | --- | --- |
|  |  | **0-2 years** | | | **3-6 years** | | |
|  |  | **N = 32 pairs** | | | **N = 32 pairs** | | |
|  | **N = 191** | **PA** | **Controls** | **Cost difference** | **PA** | **Controls** | **Cost difference** |
| **All-cause healthcare costs, mean ± SD (USD/year)** | | | | |  |  |  |
| Total medical and pharmacy costs | 88,523 ± 267,258 | 209,332 ± 343,355 | 3,449 ± 5,270 | **$205,883 ± 60,704** | 92,073 ± 145,232 | 1,294 ± 1,758 | **$90,779 ± 25,676** |
| Total medical costs | 60,385 ± 136,310 | 174,694 ± 282,715 | 3,316 ± 5,219 | **$171,377 ± 49,986** | 76,860 ± 127,501 | 1,199 ± 1,661 | **$75,661 ± 22,541** |
| IP | 40,621 ± 114,420 | 137,474 ± 246,042 | 428 ± 862 | **$137,046 ± 43,495** | 41,947 ± 87,250 | 273 ± 1,106 | **$41,674 ± 15,425** |
| ED | 3,514 ± 11,945 | 2,258 ± 3,127 | 265 ± 486 | **$1,994 ± 559** | 5,995 ± 23,677 | 83 ± 203 | **$5,911 ± 4,186** |
| HH | 6,189 ± 16,914 | 13,977 ± 24,826 | 74 ± 185 | **$13,903 ± 4,389** | 13,753 ± 46,297 | 45 ± 256 | **$13,708 ± 8,184** |
| OP | 10,053 ± 21,702 | 20,985 ± 34,467 | 2,550 ± 4,929 | **$18,435 ± 6,155** | 15,144 ± 23,372 | 798 ± 867 | **$14,347 ± 4,134** |
| SNF | 8 ± 69 | 0 ± 0 | 0 ± 0 | $0 ± 0 | 21 ± 120 | 0 ± 0 | $21 ± 21 |
| Total pharmacy costs | 28,138 ± 218,121 | 34,638 ± 112,360 | 133 ± 494 | **$34,506 ± 19,863** | 15,213 ± 78,939 | 95 ± 336 | **$15,118 ± 13,955** |
| **PA-related healthcare costs, mean ± SD (USD/year)** | | | | |  |  |  |
| Total medical costs | 38,724 ± 123,248 | 145,235 ± 272,189 | - |  | 61,108 ± 115,668 | - |  |
| IP | 33,575 ± 112,079 | 129,945 ± 247,305 | - |  | 41,116 ± 84,601 | - |  |
| ED | 1,523 ± 9,320 | 1,516 ± 2,976 | - |  | 5,215 ± 22,319 | - |  |
| HH | 1,255 ± 11,606 | 3,644 ± 15,061 | - |  | 8,440 ± 42,810 | - |  |
| OP | 2,371 ± 9,816 | 10,130 ± 27,344 | - |  | 6,337 ± 15,787 | - |  |
| SNF | 0 ± 0 | 0 ± 0 | - |  | 0 ± 0 | - |  |

**(B)**

|  | **Age strata (years)** | | | | | | | | |
| --- | --- | --- | --- | --- | --- | --- | --- | --- | --- |
|  | **7-12 years** | | | **13-17 years** | | | **18+ years** | | |
|  | **N = 36 pairs** | | | **N = 24 pairs** | | | **N = 106 pairs** | | |
|  | **PA** | **Controls** | **Cost difference** | **PA** | **Controls** | **Cost difference** | **PA** | **Controls** | **Cost difference** |
| **All-cause healthcare costs, mean ± SD (USD/year)** | | | |  |  |  |  |  |  |
| Total medical and pharmacy costs | 21,797 ± 28,176 | 1,628 ± 2,641 | **$20,168 ± 4,717** | 77,562 ± 167,300 | 2,103 ± 4,379 | **$75,459 ± 34,162** | 79,336 ± 318,845 | 4,241 ± 7,970 | **$75,096 ± 30,979** |
| Total medical costs | 18,863 ± 26,478 | 1,550 ± 2,601 | **$17,314 ± 4,434** | 27,741 ± 40,548 | 1,046 ± 1,043 | **$26,695 ± 8,280** | 43,210 ± 84,057 | 3,460 ± 7,520 | **$39,750 ± 8,197** |
| IP | 7,154 ± 21,355 | 189 ± 1,132 | **$6,966 ± 3,564** | 12,972 ± 29,796 | 0 ± 0 | **$12,972 ± 6,082** | 27,881 ± 67,783 | 1,020 ± 5,159 | **$26,861 ± 6,603** |
| ED | 1,044 ± 2,097 | 209 ± 705 | **$835 ± 369** | 2,017 ± 3,771 | 87 ± 299 | **$1,930 ± 772** | 3,547 ± 9,204 | 468 ± 1,240 | **$3,079 ± 902** |
| HH | 5,618 ± 11,194 | 33 ± 116 | **$5,585 ± 1,866** | 8,084 ± 14,485 | 175 ± 800 | **$7,910 ± 2,961** | 2,770 ± 6,043 | 42 ± 177 | **$2,727 ± 587** |
| OP | 5,047 ± 4,763 | 1,119 ± 1,924 | **$3,929 ± 856** | 4,667 ± 4,105 | 784 ± 836 | **$3,883 ± 855** | 9,003 ± 23,044 | 1,928 ± 3,761 | **$7,075 ± 2,268** |
| SNF | 0 ± 0 | 0 ± 0 | $0 ± 0 | 0 ± 0 | 0 ± 0 | $0 ± 0 | 9 ± 66 | 2 ± 19 | $7 ± 7 |
| Total pharmacy costs | 2,933 ± 7,146 | 79 ± 156 | **$2,855 ± 1,191** | 49,821 ± 152,182 | 1,057 ± 4,330 | **$48,764 ± 31,077** | 36,126 ± 306,390 | 780 ± 2,462 | **$35,346 ± 29,760** |
| **PA-related healthcare costs, mean ± SD (USD/year)** | | | |  |  |  |  |  |  |
| Total medical costs | 10,521 ± 22,907 | - |  | 16,632 ± 31,469 | - |  | 18,599 ± 60,976 | - |  |
| IP | 7,064 ± 21,379 | - |  | 12,972 ± 29,796 | - |  | 17,516 ± 60,369 | - |  |
| ED | 675 ± 1,648 | - |  | 1,066 ± 2,205 | - |  | 559 ± 1,316 | - |  |
| HH | 669 ± 2,297 | - |  | 710 ± 2,454 | - |  | 94 ± 410 | - |  |
| OP | 2,113 ± 2,745 | - |  | 1,885 ± 2,489 | - |  | 430 ± 1,094 | - |  |
| SNF | 0 ± 0 | - |  | 0 ± 0 | - |  | 0 ± 0 | - |  |

Values in boldface indicate statistically significant difference between patients with PA and control individuals within that age stratum (p<0.05).

**Abbreviations:** ED, emergency department; HH, home health; IP, inpatient; OP, outpatient; PA, propionic acidemia; SD, standard deviation; SNF, skilled nursing facility; USD, United States dollar.

**Note:**

[1] Costs were inflation-adjusted to 2022 US dollars based on the medical care component of the Consumer Price Index.

## Supplemental Table 2. Rates of all-cause and PA-related HRU among patients with PA, with and without MDEs, in the (A) 0-2 and 3-6 years age strata and (B) 7-12, 13-17, and 18+ years age strata

**(A)**

|  | **All patients with PA** | | | **Age strata (years)** | | | | | |
| --- | --- | --- | --- | --- | --- | --- | --- | --- | --- |
|  |  |  |  | **0-2 years** | | | **3-6 years** | | |
|  | **With MDE** | **Without MDE** | **RR (95% CI)** | **With MDE** | **Without MDE** | **RR (95% CI)** | **With MDE** | **Without MDE** | **RR (95% CI)** |
|  | **N = 60** | **N = 131** |  | **N = 16** | **N = 16** |  | **N = 14** | **N = 18** |  |
| **All-cause HRU, PPY (95% CI)** | | | |  |  |  |  |  |  |
| IP | 2.34 (2.13, 2.57) | 0.36 (0.30, 0.43) | **6.59 (5.39, 8.14)** | 3.20 (2.63, 3.89) | 0.26 (0.13, 0.52) | **12.33 (6.41, 27.57)** | 3.86 (3.27, 4.55) | 0.25 (0.12, 0.53) | **15.19 (7.68, 35.84)** |
| ED | 2.12 (1.92, 2.34) | 1.05 (0.95, 1.17) | **2.01 (1.74, 2.32)** | 1.89 (1.46, 2.43) | 1.07 (0.76, 1.50) | **1.76 (1.16, 2.73)** | 1.74 (1.36, 2.22) | 0.83 (0.55, 1.26) | **2.08 (1.31, 3.42)** |
| HH | 28.84 (28.08, 29.63) | 5.55 (5.30, 5.82) | **5.19 (4.92, 5.48)** | 38.62 (36.50, 40.86) | 7.23 (6.34, 8.24) | **5.35 (4.64, 6.18)** | 39.70 (37.72, 41.79) | 4.61 (3.87, 5.48) | **8.62 (7.22, 10.38)** |
| OP | 37.41 (36.54, 38.29) | 16.65 (16.21, 17.10) | **2.25 (2.17, 2.33)** | 48.82 (46.43, 51.33) | 17.40 (15.99, 18.93) | **2.81 (2.55, 3.10)** | 45.75 (43.62, 47.99) | 35.44 (33.28, 37.73) | **1.29 (1.19, 1.40)** |
| **PA-related HRU, PPY (95% CI)** | | | |  |  |  |  |  |  |
| IP | 1.88 (1.69, 2.09) | 0.12 (0.08, 0.16) | **16.31 (11.79, 23.27)** | 2.88 (2.34, 3.54) | 0.10 (0.03, 0.30) | **29.60 (11.12, 120.52)** | 3.72 (3.15, 4.40) | 0.25 (0.12, 0.53) | **14.65 (7.40, 34.59)** |
| ED | 0.64 (0.53, 0.77) | 0.20 (0.16, 0.25) | **3.21 (2.38, 4.37)** | 1.25 (0.91, 1.71) | 0.16 (0.07, 0.39) | **7.70 (3.33, 22.33)** | 0.81 (0.57, 1.17) | 0.51 (0.30, 0.86) | 1.60 (0.87, 3.12) |
| HH | 3.30 (3.05, 3.57) | 0.31 (0.25, 0.37) | **10.80 (8.77, 13.45)** | 7.16 (6.28, 8.16) | - | - | 8.96 (8.04, 9.98) | 0.29 (0.15, 0.58) | **30.88 (16.42, 68.02)** |
| OP | 7.56 (7.18, 7.97) | 2.25 (2.09, 2.42) | **3.36 (3.07, 3.68)** | 21.07 (19.52, 22.74) | 2.59 (2.08, 3.23) | **8.13 (6.49, 10.33)** | 12.63 (11.53, 13.83) | 5.91 (5.07, 6.89) | **2.14 (1.79, 2.56)** |

**(B)**

|  | **Age strata (years)** | | | | | | | | |
| --- | --- | --- | --- | --- | --- | --- | --- | --- | --- |
|  | **7-12 years** | | | **13-17 years** | | | **18+ years** | | |
|  | **With MDE** | **Without MDE** | **RR (95% CI)** | **With MDE** | **Without MDE** | **RR (95% CI)** | **With MDE** | **Without MDE** | **RR (95% CI)** |
|  | **N = 9** | **N = 27** |  | **N = 6** | **N = 18** |  | **N = 34** | **N = 72** |  |
| **All-cause HRU, PPY (95% CI)** | | | |  |  |  |  |  |  |
| IP | 1.36 (0.97, 1.90) | 0.13 (0.07, 0.26) | **10.15 (5.09, 22.53)** | 1.99 (1.30, 3.06) | 0.10 (0.03, 0.30) | **20.93 (7.22, 88.61)** | 1.69 (1.43, 1.99) | 0.53 (0.43, 0.66) | **3.17 (2.43, 4.16)** |
| ED | 0.92 (0.61, 1.38) | 0.58 (0.42, 0.79) | 1.58 (0.93, 2.63) | 1.52 (0.93, 2.48) | 0.48 (0.29, 0.79) | **3.19 (1.57, 6.52)** | 2.81 (2.47, 3.20) | 1.39 (1.22, 1.59) | **2.02 (1.68, 2.43)** |
| HH | 18.96 (17.33, 20.75) | 5.18 (4.66, 5.75) | **3.66 (3.19, 4.21)** | 51.26 (47.11, 55.77) | 16.35 (15.00, 17.83) | **3.13 (2.78, 3.54)** | 20.42 (19.46, 21.42) | 3.48 (3.20, 3.77) | **5.87 (5.35, 6.47)** |
| OP | 20.92 (19.20, 22.80) | 13.25 (12.41, 14.15) | **1.58 (1.42, 1.76)** | 31.61 (28.39, 35.19) | 16.19 (14.85, 17.66) | **1.95 (1.70, 2.24)** | 35.08 (33.83, 36.38) | 14.82 (14.25, 15.43) | **2.37 (2.24, 2.50)** |
| **PA-related HRU, PPY (95% CI)** | | | |  |  |  |  |  |  |
| IP | 1.36 (0.97, 1.90) | 0.12 (0.06, 0.24) | **11.42 (5.57, 26.54)** | 1.99 (1.30, 3.06) | 0.10 (0.03, 0.30) | **20.93 (7.22, 88.61)** | 0.82 (0.65, 1.05) | 0.10 (0.06, 0.16) | **8.44 (5.03, 15.06)** |
| ED | 0.32 (0.16, 0.64) | 0.22 (0.13, 0.37) | 1.43 (0.58, 3.30) | 1.14 (0.65, 2.01) | 0.13 (0.05, 0.34) | **8.97 (3.12, 32.08)** | 0.36 (0.25, 0.52) | 0.16 (0.11, 0.23) | **2.29 (1.36, 3.90)** |
| HH | 0.92 (0.61, 1.38) | 0.28 (0.18, 0.44) | **3.25 (1.77, 6.04)** | 1.14 (0.65, 2.01) | 1.71 (1.31, 2.24) | 0.66 (0.34, 1.20) | 0.30 (0.20, 0.45) | 0.10 (0.06, 0.17) | **2.92 (1.59, 5.50)** |
| OP | 4.12 (3.40, 5.00) | 2.62 (2.26, 3.04) | **1.57 (1.23, 2.00)** | 7.78 (6.27, 9.66) | 4.22 (3.56, 5.00) | **1.84 (1.39, 2.42)** | 1.19 (0.98, 1.45) | 1.04 (0.89, 1.21) | 1.14 (0.89, 1.46) |

Values in boldface indicate statistically significant difference between patients with PA and MDEs and patients with PA without MDEs within that age stratum (p<0.05).

**Abbreviations:** CI, confidence interval; ED, emergency department; HH, home health; HRU, healthcare resource utilization; IP, inpatient; MDE, metabolic decompensation event; OP, outpatient; PA, propionic acidemia; PPY, per-person-year; RR, rate ratio.

## Supplemental Table 3. Detailed annualized all-cause and PA-related healthcare costs among patients with PA, with and without MDEs, in the (A) 0-2 and 3-6 years age strata and (B) 7-12, 13-17, and 18+ years age strata

**(A)**

|  |  |  |  | **Age strata (years)** | | | | | |
| --- | --- | --- | --- | --- | --- | --- | --- | --- | --- |
|  | **All patients with PA** | | | **0-2 years** | | | **3-6 years** | | |
|  | **With MDE** | **Without MDE** | **Cost difference** | **With MDE** | **Without MDE** | **Cost difference** | **With MDE** | **Without MDE** | **Cost difference** |
|  | **N = 60** | **N = 131** |  | **N = 16** | **N = 16** |  | **N = 14** | **N = 18** |  |
| **All-cause healthcare costs, mean ± SD (USD/year)** | | | |  |  |  |  |  |  |
| Total medical and pharmacy costs | 227,184 ± 441,613 | 25,014 ± 54,268 | **202,171 ± 57,209** | 389,234 ± 413,837 | 29,430 ± 57,778 | **359,805 ± 104,463** | 154,661 ± 156,143 | 43,393 ± 118,673 | **111,268 ± 50,238** |
| Total medical costs | 143,755 ± 208,421 | 22,201 ± 52,572 | **121,554 ± 27,296** | 320,984 ± 340,977 | 28,403 ± 57,125 | **292,581 ± 86,432** | 120,770 ± 130,744 | 42,707 ± 117,257 | 78,063 ± 44,552 |
| IP | 106,958 ± 181,940 | 10,238 ± 33,907 | **96,721 ± 23,674** | 266,486 ± 298,230 | 8,461 ± 25,692 | **258,025 ± 74,834** | 70,624 ± 96,411 | 19,642 ± 74,630 | 50,982 ± 31,199 |
| ED | 5,191 ± 9,309 | 2,745 ± 12,935 | 2,445 ± 1,650 | 3,147 ± 3,714 | 1,370 ± 2,174 | 1,777 ± 1,076 | 2,303 ± 2,914 | 8,866 ± 31,557 | -6,563 ± 7,479 |
| HH | 12,474 ± 24,772 | 3,311 ± 10,633 | **9,163 ± 3,330** | 21,246 ± 24,545 | 6,708 ± 23,634 | 14,538 ± 8,518 | 28,514 ± 68,222 | 2,272 ± 5,516 | 26,242 ± 18,279 |
| OP | 19,117 ± 33,817 | 5,901 ± 10,679 | **13,216 ± 4,464** | 30,106 ± 42,722 | 11,864 ± 21,273 | 18,242 ± 11,931 | 19,330 ± 26,426 | 11,889 ± 20,891 | 7,441 ± 8,610 |
| SNF | 15 ± 87 | 6 ± 60 | 9 ± 12 | 0 ± 0 | 0 ± 0 | 0 ± 0 | 0 ± 0 | 38 ± 160 | -38 ± 38 |
| Total pharmacy costs | 83,430 ± 385,448 | 2,813 ± 7,071 | **80,617 ± 49,765** | 68,250 ± 153,872 | 1,027 ± 2,132 | 67,223 ± 38,472 | 33,891 ± 119,112 | 686 ± 1,676 | 33,205 ± 31,836 |
| **PA-related healthcare costs, mean ± SD (USD/year)** | | | |  |  |  |  |  |  |
| Total medical costs | 107,047 ± 195,932 | 7,430 ± 40,457 | **99,617 ± 25,540** | 288,397 ± 330,727 | 2,073 ± 3,010 | **286,324 ± 82,685** | 99,369 ± 119,061 | 31,349 ± 106,836 | 68,021 ± 40,579 |
| IP | 96,310 ± 181,182 | 4,841 ± 28,508 | **91,469 ± 23,523** | 259,309 ± 301,151 | 581 ± 1,855 | **258,728 ± 75,289** | 68,724 ± 91,239 | 19,642 ± 74,630 | 49,082 ± 30,067 |
| ED | 1,625 ± 2,700 | 1,476 ± 11,120 | 148 ± 1,032 | 2,613 ± 3,790 | 419 ± 1,171 | **2,194 ± 992** | 1,269 ± 2,165 | 8,285 ± 29,697 | -7,017 ± 7,024 |
| HH | 3,611 ± 20,588 | 176 ± 868 | 3,435 ± 2,659 | 7,288 ± 20,987 | 0 ± 0 | 7,288 ± 5,247 | 18,862 ± 64,503 | 334 ± 1,105 | 18,529 ± 17,241 |
| OP | 5,502 ± 16,965 | 936 ± 1,898 | **4,566 ± 2,196** | 19,187 ± 36,978 | 1,073 ± 1,677 | 18,114 ± 9,254 | 10,514 ± 23,311 | 3,088 ± 3,663 | 7,426 ± 6,290 |
| SNF | 0 ± 0 | 0 ± 0 | 0 ± 0 | 0 ± 0 | 0 ± 0 | 0 ± 0 | 0 ± 0 | 0 ± 0 | 0 ± 0 |

**(B)**

|  | **Age strata (years)** | | | | | | | | |
| --- | --- | --- | --- | --- | --- | --- | --- | --- | --- |
|  | **7-12 years** | | | **13-17 years** | | | **18+ years** | | |
|  | **With MDE** | **Without MDE** | **Cost difference** | **With MDE** | **Without MDE** | **Cost difference** | **With MDE** | **Without MDE** | **Cost difference** |
|  | **N = 9** | **N = 27** |  | **N = 6** | **N = 18** |  | **N = 34** | **N = 72** |  |
| **All-cause healthcare costs, mean ± SD (USD/year)** | | | |  |  |  |  |  |  |
| Total medical and pharmacy costs | 43,353 ± 42,790 | 14,611 ± 17,053 | **28,742 ± 14,636** | 274,228 ± 257,593 | 12,006 ± 12,271 | **262,221 ± 105,202** | 197,633 ± 547,535 | 23,474 ± 33,736 | **174,159 ± 93,986** |
| Total medical costs | 40,147 ± 43,417 | 11,769 ± 12,438 | **28,378 ± 14,669** | 81,248 ± 50,485 | 9,905 ± 11,289 | **71,343 ± 20,782** | 92,568 ± 129,626 | 19,902 ± 30,359 | **72,667 ± 22,517** |
| IP | 24,653 ± 38,833 | 1,322 ± 2,932 | **23,332 ± 12,957** | 47,204 ± 46,383 | 1,561 ± 4,114 | **45,642 ± 18,960** | 63,292 ± 107,092 | 11,159 ± 24,085 | **52,133 ± 18,584** |
| ED | 1,341 ± 2,321 | 944 ± 2,054 | 397 ± 869 | 4,678 ± 5,629 | 1,131 ± 2,567 | **3,548 ± 2,376** | 6,510 ± 11,790 | 2,148 ± 7,382 | **4,362 ± 2,201** |
| HH | 7,280 ± 8,771 | 5,064 ± 11,989 | 2,216 ± 3,725 | 21,025 ± 20,380 | 3,770 ± 9,105 | **17,254 ± 8,593** | 4,932 ± 8,541 | 1,749 ± 4,098 | **3,184 ± 1,542** |
| OP | 6,872 ± 5,249 | 4,439 ± 4,531 | 2,433 ± 1,955 | 8,342 ± 4,438 | 3,443 ± 3,265 | **4,899 ± 1,968** | 17,808 ± 37,951 | 4,845 ± 7,822 | **12,963 ± 6,574** |
| SNF | 0 ± 0 | 0 ± 0 | 0 ± 0 | 0 ± 0 | 0 ± 0 | 0 ± 0 | 26 ± 115 | 1 ± 6 | 25 ± 20 |
| Total pharmacy costs | 3,206 ± 5,914 | 2,842 ± 7,612 | 364 ± 2,456 | 192,979 ± 271,464 | 2,101 ± 3,797 | **190,878 ± 110,828** | 105,065 ± 539,761 | 3,572 ± 8,116 | 101,492 ± 92,573 |
| **PA-related healthcare costs, mean ± SD (USD/year)** | | | |  |  |  |  |  |  |
| Total medical costs | 30,498 ± 40,356 | 3,862 ± 4,594 | **26,636 ± 13,481** | 54,360 ± 46,706 | 4,056 ± 5,323 | **50,304 ± 19,109** | 49,580 ± 100,914 | 3,969 ± 9,411 | **45,611 ± 17,342** |
| IP | 24,653 ± 38,833 | 1,201 ± 2,917 | **23,452 ± 12,956** | 47,204 ± 46,383 | 1,561 ± 4,114 | **45,642 ± 18,960** | 47,967 ± 100,043 | 3,137 ± 9,170 | **44,829 ± 17,191** |
| ED | 859 ± 1,669 | 614 ± 1,669 | 245 ± 642 | 2,814 ± 3,231 | 484 ± 1,439 | **2,330 ± 1,362** | 895 ± 1,719 | 400 ± 1,052 | 496 ± 320 |
| HH | 2,036 ± 4,218 | 214 ± 874 | 1,822 ± 1,416 | 843 ± 1,041 | 665 ± 2,797 | 178 ± 784 | 114 ± 392 | 84 ± 421 | 30 ± 84 |
| OP | 2,950 ± 2,946 | 1,834 ± 2,674 | 1,116 ± 1,109 | 3,500 ± 2,836 | 1,346 ± 2,188 | 2,154 ± 1,267 | 603 ± 1,629 | 348 ± 718 | 256 ± 292 |
| SNF | 0 ± 0 | 0 ± 0 | 0 ± 0 | 0 ± 0 | 0 ± 0 | 0 ± 0 | 0 ± 0 | 0 ± 0 | 0 ± 0 |

Values in boldface indicate statistically significant difference between patients with PA and MDEs and patients with PA without MDEs within that age stratum (p<0.05).

**Abbreviations:** ED, emergency department; HH, home health; IP, inpatient; MDE, metabolic decompensation event; OP, outpatient; PA, propionic acidemia; SD, standard deviation; SNF, skilled nursing facility; USD, United States dollar.

## Supplemental Table 4. RRs of all-cause HRU among patients with PA, with or without MDEs, versus matched non-PA control individuals

| **All-cause HRU, PPY, RR (95% CI)** | **Age strata (years)** | | | | |
| --- | --- | --- | --- | --- | --- |
|  | **0-2 years** | **3-6 years** | **7-12 years** | **13-17 years** | **18+ years** |
| **Patients with PA, with MDEs, versus matched non-PA control individuals** | | | | | |
| **Number of patients** | **N = 16 pairs** | **N = 14 pairs** | **N = 9 pairs** | **N = 6 pairs** | **N = 34 pairs** |
| IP | **18.71 (8.96, 47.93)** | **132.71 (29.81, 2334.78)** | - | - | **45.46 (19.20, 147.91)** |
| OP | **5.99 (5.29, 6.81)** | **8.08 (6.98, 9.39)** | **5.19 (4.29, 6.32)** | **3.58 (2.86, 4.53)** | **4.64 (4.29, 5.01)** |
| HH | **169.54 (90.83, 371.67)** | **56.93 (38.98, 87.65)** | - | **33.76 (21.28, 57.91)** | **57.94 (42.67, 81.31)** |
| SNF | - | - | - | - | - |
| ED | **4.73 (2.72, 8.82)** | **2.72 (1.70, 4.51)** | **3.62 (1.69, 8.64)** | **5.33 (1.78, 22.94)** | **7.78 (5.61, 11.09)** |
| **Patients with PA, without MDEs, versus matched non-PA control individuals** | | | | | |
| **Number of patients** | **N = 16 pairs** | **N = 18 pairs** | **N = 27 pairs** | **N = 18 pairs** | **N = 72 pairs** |
| IP | 1.57 (0.55, 4.78) | **8.50 (1.51, 158.89)** | **10.62 (2.00, 195.79)** | - | **10.62 (5.80, 21.79)** |
| OP | **1.60 (1.41, 1.82)** | **8.07 (6.81, 9.64)** | **1.36 (1.24, 1.50)** | **2.99 (2.53, 3.54)** | **1.72 (1.62, 1.83)** |
| HH | **43.89 (21.37, 111.33)** | - | **37.32 (21.55, 72.42)** | - | **15.09 (11.31, 20.66)** |
| SNF | - | - | - | - | 1.22 (0.05, 30.86) |
| ED | 1.62 (0.96, 2.78) | **27.93 (5.89, 499.76)** | **2.00 (1.21, 3.40)** | 1.38 (0.65, 3.02) | **3.09 (2.43, 3.96)** |

Values in boldface indicate statistically significant difference between patients with PA and control individuals within that age stratum (p<0.05).

**Abbreviations:** CI, confidence interval; ED, emergency department; HH, home health; IP, inpatient; MDE, metabolic decompensation event; OP, outpatient; PA, propionic acidemia; PPY, per-person-year; RR, rate ratio; SNF, skilled nursing facility.

## Supplemental Table 5. Cost differences of annualized all-cause healthcare costs among patients with PA, with or without MDEs, versus matched non-PA control individuals

| **All-cause healthcare cost difference, mean ± SD (USD/year)** | **Age strata (years)** | | | | |
| --- | --- | --- | --- | --- | --- |
|  | **0-2 years** | **3-6 years** | **7-12 years** | **13-17 years** | **18+ years** |
| **Patients with PA, with MDEs, versus matched non-PA control individuals** | | | | | |
| **Number of patients** | **N = 16 pairs** | **N = 14 pairs** | **N = 9 pairs** | **N = 6 pairs** | **N = 34 pairs** |
| **Total medical and pharmacy costs** | **386,700 ± 103,460** | **152,839 ± 41,735** | **42,873 ± 14,264** | **268,403 ± 105,213** | **194,063 ± 93,907** |
| **Total medical costs** | **318,631 ± 85,245** | **118,959 ± 34,948** | **39,702 ± 14,473** | **79,055 ± 20,619** | **90,151 ± 22,243** |
| IP | **266,105 ± 74,558** | **70,237 ± 25,770** | **24,653 ± 12,944** | 47,204 ± 18,936 | **62,628 ± 18,372** |
| OP | **28,520 ± 10,683** | **18,199 ± 7,070** | **6,489 ± 1,752** | **7,089 ± 1,907** | **16,424 ± 6,515** |
| HH | **21,144 ± 6,136** | **28,411 ± 18,233** | **7,280 ± 2,924** | **20,327 ± 8,345** | **4,854 ± 1,466** |
| SNF | 0 ± 0 | 0 ± 0 | 0 ± 0 | 0 ± 0 | 26 ± 20 |
| ED | **2,862 ± 940** | **2,112 ± 782** | **1,279 ± 775** | 4,436 ± 2,311 | **6,219 ± 2,025** |
| **Total pharmacy costs** | **68,069 ± 38,468** | **33,880 ± 31,834** | 3,171 ± 1,971 | **189,348 ± 110,881** | **103,912 ± 92,569** |
| **Patients with PA, without MDEs, versus matched non-PA control individuals** | | | | | |
| **Number of patients** | **N = 16 pairs** | **N = 18 pairs** | **N = 27 pairs** | **N = 18 pairs** | **N = 72 pairs** |
| **Total medical and pharmacy costs** | **25,066 ± 14,559** | **42,510 ± 27,973** | **12,600 ± 3,331** | **11,145 ± 2,896** | **18,916 ± 4,111** |
| **Total medical costs** | **24,123 ± 14,394** | **41,984 ± 27,639** | **9,851 ± 2,459** | **9,242 ± 2,663** | **15,949 ± 3,719** |
| IP | 7,986 ± 6,427 | 19,458 ± 17,591 | **1,070 ± 618** | 1,561 ± 970 | **9,972 ± 2,925** |
| OP | **8,349 ± 5,589** | **11,351 ± 4,925** | **3,075 ± 967** | **2,815 ± 777** | **2,660 ± 1,058** |
| HH | 6,662 ± 5,909 | **2,272 ± 1,300** | **5,020 ± 2,307** | **3,770 ± 2,146** | **1,723 ± 483** |
| SNF | 0 ± 0 | 38 ± 38 | 0 ± 0 | 0 ± 0 | -2 ± 3 |
| ED | 1,125 ± 552 | **8,866 ± 7,438** | 686 ± 425 | 1,095 ± 605 | **1,596 ± 886** |
| **Total pharmacy costs** | 943 ± 536 | 526 ± 409 | **2,749 ± 1,465** | 1,903 ± 898 | **2,967 ± 993** |

Values in boldface indicate statistically significant difference between patients with PA and control individuals within that age stratum (p<0.05).

**Abbreviations:** ED, emergency department; HH, home health; IP, inpatient; MDE, metabolic decompensation event; OP, outpatient; PA, propionic acidemia; SD, standard deviation; SNF, skilled nursing facility; USD, United States dollar.

## Supplemental Table 6. RRs of all-cause and PA-related HRU among patients with PA, pre- versus post COVID-19 onset

|  |  | **Age strata (years)** | | | | |
| --- | --- | --- | --- | --- | --- | --- |
|  | **All patients with PA** | **0-2 years** | **3-6 years** | **7-12 years** | **13-17 years** | **18+ years** |
|  | Pre-COVID-19  (N = 140) vs. | Pre-COVID-19  (N = 24) vs. | Pre-COVID-19  (N = 22) vs. | Pre-COVID-19  (N = 29) vs. | Pre-COVID-19  (N = 16) vs. | Pre-COVID-19 (N = 73) vs. |
|  | Post COVID-19  (N = 119) | Post COVID-19  (N = 13) | Post COVID-19  (N = 14) | Post COVID-19  (N = 18) | Post COVID-19  (N = 10) | Post COVID-19 (N = 70) |
| **All-cause HRU, RR (95% CI)** | | | | | | |
| IP | **1.72 (1.43, 2.08)** | 1.09 (0.72, 1.69) | **2.91 (1.93, 4.54)** | **0.33 (0.17, 0.60)** | **6.45 (1.90, 40.29)** | **1.95 (1.47, 2.61)** |
| OP | **1.10 (1.06, 1.14)** | **1.16 (1.06, 1.28)** | **0.86 (0.80, 0.93)** | **1.13 (1.01, 1.27)** | **1.63 (1.40, 1.90)** | **1.10 (1.04, 1.16)** |
| HH | **1.18 (1.12, 1.24)** | 1.04 (0.92, 1.17) | **0.28 (0.25, 0.31)** | **1.41 (1.21, 1.65)** | **7.24 (5.80, 9.18)** | **1.63 (1.50, 1.78)** |
| ED | **1.35 (1.16, 1.57)** | 1.20 (0.76, 1.95) | 1.45 (0.93, 2.34) | **2.09 (1.15, 4.11)** | 2.01 (0.91, 5.06) | **1.41 (1.17, 1.70)** |
| **PA-related HRU, RR (95% CI)** | | | | | | |
| IP | **2.00 (1.59, 2.53)** | 1.35 (0.85, 2.23) | **3.07 (2.01, 4.88)** | **0.34 (0.18, 0.63)** | **6.45 (1.90, 40.29)** | **2.27 (1.41, 3.79)** |
| OP | **1.73 (1.58, 1.91)** | **2.82 (2.29, 3.52)** | **0.61 (0.52, 0.71)** | **1.48 (1.13, 1.95)** | **3.70 (2.55, 5.59)** | **2.00 (1.55, 2.62)** |
| HH | **0.50 (0.43, 0.58)** | **1.49 (1.03, 2.20)** | **0.04 (0.03, 0.06)** | **2.51 (1.18, 6.16)** | **38.14 (8.44, 673.69)** | **2.43 (1.26, 5.05)** |
| ED | **1.88 (1.36, 2.65)** | 1.25 (0.65, 2.60) | 1.33 (0.71, 2.59) | **5.27 (1.55, 32.93)** | 4.11 (1.15, 26.16) | 1.68 (0.96, 3.02) |

Values in boldface indicate statistically significant difference between patients with PA during the pre-COVID-19 vs. post COVID-19 period within that age stratum (p<0.05).

**Abbreviations:** CI, confidence interval; ED, emergency department; HH, home health; HRU, healthcare resource utilization; IP, inpatient; OP, outpatient; PA, propionic acidemia; RR, rate ratio.

## Supplemental Table 7. Cost differences of annualized all-cause healthcare costs and PA-related medical costs among patients with PA, pre- versus post COVID-19 onset

|  |  | **Age strata (years)** | | | | |
| --- | --- | --- | --- | --- | --- | --- |
|  | **All patients with PA** | **0-2 years** | **3-6 years** | **7-12 years** | **13-17 years** | **18+ years** |
|  | Pre-COVID-19  (N = 140) vs. | Pre-COVID-19  (N = 24) vs. | Pre-COVID-19  (N = 22) vs. | Pre-COVID-19  (N = 29) vs. | Pre-COVID-19  (N = 16) vs. | Pre-COVID-19  (N = 73) vs. |
|  | Post COVID-19 (N = 119) | Post COVID-19 (N = 13) | Post COVID-19 (N = 14) | Post COVID-19 (N = 18) | Post COVID-19 (N = 10) | Post COVID-19 (N = 70) |
| **All-cause healthcare cost difference, mean ± SD (USD/year)** | | | | | | |
| **Total medical and pharmacy costs** | $4,038 ± 39,122 | $-49,764 ± 141,011 | $-56,473 ± 57,926 | $-1,618 ± 8,876 | $103,266 ± 49,368 | $17,075 ± 63,708 |
| **Total medical costs** | $13,133 ± 19,413 | $11,118 ± 111,154 | $-19,907 ± 49,522 | $-2,898 ± 8,484 | $29,247 ± 11,810 | $8,641 ± 15,717 |
| IP | $8,785 ± 16,529 | $11,127 ± 101,565 | $-1,287 ± 37,521 | $-6,365 ± 7,044 | $15,493 ± 9,056 | $2,215 ± 12,829 |
| OP | $3,250 ± 2,822 | $6,327 ± 9,951 | $-3,903 ± 8,411 | $1,174 ± 1,536 | $3,035 ± 1,486 | $3,451 ± 3,765 |
| HH | $-744 ± 2,656 | $-7,574 ± 8,465 | $-21,115 ± 18,466 | $1,110 ± 2,700 | $9,187 ± 4,315 | $2,088 ± 1,182 |
| SNF | $-4 ± 8 | $0 ± 0 | $-49 ± 49 | $0 ± 0 | $0 ± 0 | $3 ± 11 |
| ED | $1,846 ± 1,283 | $1,238 ± 1,328 | $6,447 ± 6,126 | $1,184 ± 494 | $1,532 ± 1,437 | $884 ± 1,497 |
| **Total pharmacy costs** | $-9,095 ± 32,721 | $-60,882 ± 46,791 | $-36,566 ± 35,412 | $1,279 ± 2,058 | $74,019 ± 45,766 | $8,434 ± 61,283 |
| **PA-related medical cost difference, mean ± SD (USD/year)** | | | | | | |
| **Total medical costs** | $12,656 ± 17,720 | $27,961 ± 107,170 | $-18,478 ± 45,064 | $-4,032 ± 7,474 | $18,820 ± 9,410 | $5,190 ± 11,726 |
| IP | $11,146 ± 16,229 | $15,047 ± 101,714 | $-1,617 ± 36,279 | $-6,130 ± 7,059 | $15,493 ± 9,056 | $4,695 ± 11,593 |
| OP | $1,683 ± 1,571 | $10,491 ± 7,841 | $-5,636 ± 6,385 | $721 ± 791 | $1,471 ± 1,061 | $280 ± 184 |
| HH | $-1,527 ± 2,084 | $1,371 ± 2,594 | $-17,101 ± 17,285 | $406 ± 517 | $1,048 ± 744 | $106 ± 74 |
| SNF | $0 ± 0 | $0 ± 0 | $0 ± 0 | $0 ± 0 | $0 ± 0 | $0 ± 0 |
| ED | $1,353 ± 947 | $1,052 ± 1,289 | $5,876 ± 5,781 | $971 ± 414 | $808 ± 856 | $109 ± 278 |

No statistically significant differences were observed between patients with PA during the pre-COVID-19 vs. post COVID-19 period within each age stratum (p<0.05).

**Abbreviations:** ED, emergency department; HH, home health; IP, inpatient; OP, outpatient; PA, propionic acidemia; SD, standard deviation; SNF, skilled nursing facility; USD, United States dollar.
